# Evolution of ferritin levels in hepatitis C patients treated with antivirals

**DOI:** 10.1038/s41598-020-76871-z

**Published:** 2020-11-12

**Authors:** Ming-Ling Chang, Jing-Hong Hu, Ching-Hao Yen, Kuan-Hsing Chen, Chia-Jung Kuo, Ming-Shyan Lin, Cheng-Han Lee, Shiang-Chi Chen, Rong-Nan Chien

**Affiliations:** 1 Division of Hepatology, Department of Gastroenterology and Hepatology, Liver Research Center, Chang Gung Memorial Hospital, No 5, Fu Hsing Street, Kuei Shan, Taoyuan, Taiwan; 2 Department of Medicine, College of Medicine, Chang Gung University, Taoyuan, Taiwan; 3 Division of Gastroenterology and Hepatology, Department of Internal Medicine, Chang Gung Memorial Hospital, Yunlin, Taiwan; 4 Department of Medical Education, Chang Gung Memorial Hospital, Kaohsiung, Taiwan; 5 Department of Nephrology, Kidney Research Center, Chang Gung Memorial Hospital, Linkou, Taiwan; 6 Department of Cardiology, Heart Failure Center, Chang Gung Memorial Hospital, Taoyuan, Taiwan; 7 Department of Nursing, Taipei Medical University, Taipei, Taiwan

**Keywords:** Biochemistry, Gastroenterology

## Abstract

The evolution of ferritin levels in hepatitis C virus (HCV)-infected patients with sustained virological responses (SVRs) following various therapy regimens remains elusive. An 8-year prospective cohort study of 1194 HCV-infected patients [interferon-based therapy (n = 620), direct-acting antiviral agent (DAA) therapy (n = 355)] was conducted. At baseline, sex, alanine aminotransferase (ALT), triglycerides, homeostatic model assessment of insulin resistance (HOMA-IR), estimated glomerular filtration rate (eGFR), hemoglobin, iron/total iron-binding capacity (Fe/TIBC) and IFNL3-rs12979860 genotypes were associated with ferritin levels. At 24 weeks posttherapy, ALT, triglycerides, total cholesterol, eGFR, Fe/TIBC and the therapy regimen were associated with ferritin levels in SVR patients. Among interferon-treated patients, ferritin levels increased at 24 weeks posttherapy, regardless of SVR, and 24-week posttherapy ferritin levels were higher in non-SVR patients (n = 111) than in SVR patients (n = 509); ferritin levels began decreasing at 3 years posttherapy and were lower than pretherapy levels since 4 years posttherapy in SVR patients. Among DAA-treated SVR patients (n = 350), ferritin levels decreased and remained stable since 24 weeks posttherapy. ALT, triglycerides, eGFR, and Fe/TIBC were HCV-unrelated factors associated with ferritin levels; sex, HOMA-IR, total cholesterol, hemoglobin and IFNL3-rs12979860 genotype were HCV-related factors associated with ferritin levels. In interferon-treated SVR patients, the increased trend of posttherapy ferritin levels was not reversed until 4 years posttherapy. In DAA-treated SVR patients, ferritin levels decreased since 24 weeks posttherapy.

## Introduction

Hepatitis C virus (HCV) is a human pathogen responsible for acute and chronic liver disease that chronically infects an estimated 71.1 million individuals worldwide^1^ and is classified into 8 genotypes^2^. Chronic HCV infection (CHC) is characterized by hepatic iron overload, hyperferraemia and hyperferritinaemia^3,4^, as an estimated 30–40% of CHC patients have elevated ferritin levels^5^. Ferritin keeps iron in a nontoxic form and reflects body iron stores^6^; it is also an acute phase protein and becomes elevated as inflammation occurs in chronic liver injury^7^. Transfusion (an important risk factor for HCV infection)^8^, oxidative stress^9–11^, steatosis^12^ and fibrosis^12,13^ are all associated with hyperferritinaemia in CHC patients. Iron overload with hyperferremia might suppress functions of the complement system^14^ and accelerate the persistence of HCV infection. In addition, compared with patients who respond to interferon-based anti-HCV therapy, CHC patients who do not respond had higher baseline serum ferritin levels^15,16^, and a serum ferritin level that is higher during therapy than at baseline was shown to be associated with a favorable treatment response^16^. Serum ferritin levels thus might be affected not only by HCV-related hepatic injury but also by interferon-based anti-HCV therapy, as interferon has been associated with increases in lipid levels^17^ and with immune modulation in patients^18^. With the advent of direct-acting antiviral agents (DAAs), which target specific proteins of HCV during its life cycle^19^, anti-HCV therapy has resulted in a high cure rate with a short treatment duration in CHC patients, and HCV-associated ferritin level alterations may not be masked by any interferon effect. The factors that affect the ferritin levels in CHC patients and how the anti-HCV therapeutic regimens and responses affect the serum ferritin levels remain elusive but are crucial for the prognosis of CHC patients, as ferritin is essential for iron homeostasis and is involved in a wide range of physiologic and pathologic processes^20^, and the ferritin levels in CHC patients with sustained virological response (SVR) might serve as a prognostic marker. Comparing the pre- and posttreatment variables in SVR patients has provided an excellent opportunity to eliminate the interference caused by individual bias^21^ when reviewing the impact of HCV on alterations in ferritin levels. Accordingly, we sought to fill the aforementioned knowledge gaps by conducting an 8-year prospective cohort study analyzing the serum ferritin levels and crucial confounders of CHC patients before and after interferon-based or DAA-based anti-HCV therapy.

## Materials and methods

### Patients

The study group comprised subjects aged 18 years or older with CHC, defined as serum HCV-RNA detectable by polymerase chain reaction (PCR) for > 24 weeks. Subjects with human immunodeficiency virus; hepatitis B virus infection; hemochromatosis; primary biliary cholangitis; primary sclerosing cholangitis; autoimmune hepatitis; autoimmune diseases including Sjogren's syndrome, systemic lupus erythematous, rheumatoid arthritis and psoriasis; alcoholism; or malignancy and recipients of solid organ transplants were excluded^22,23^.

### Study design

**Figure 1:**
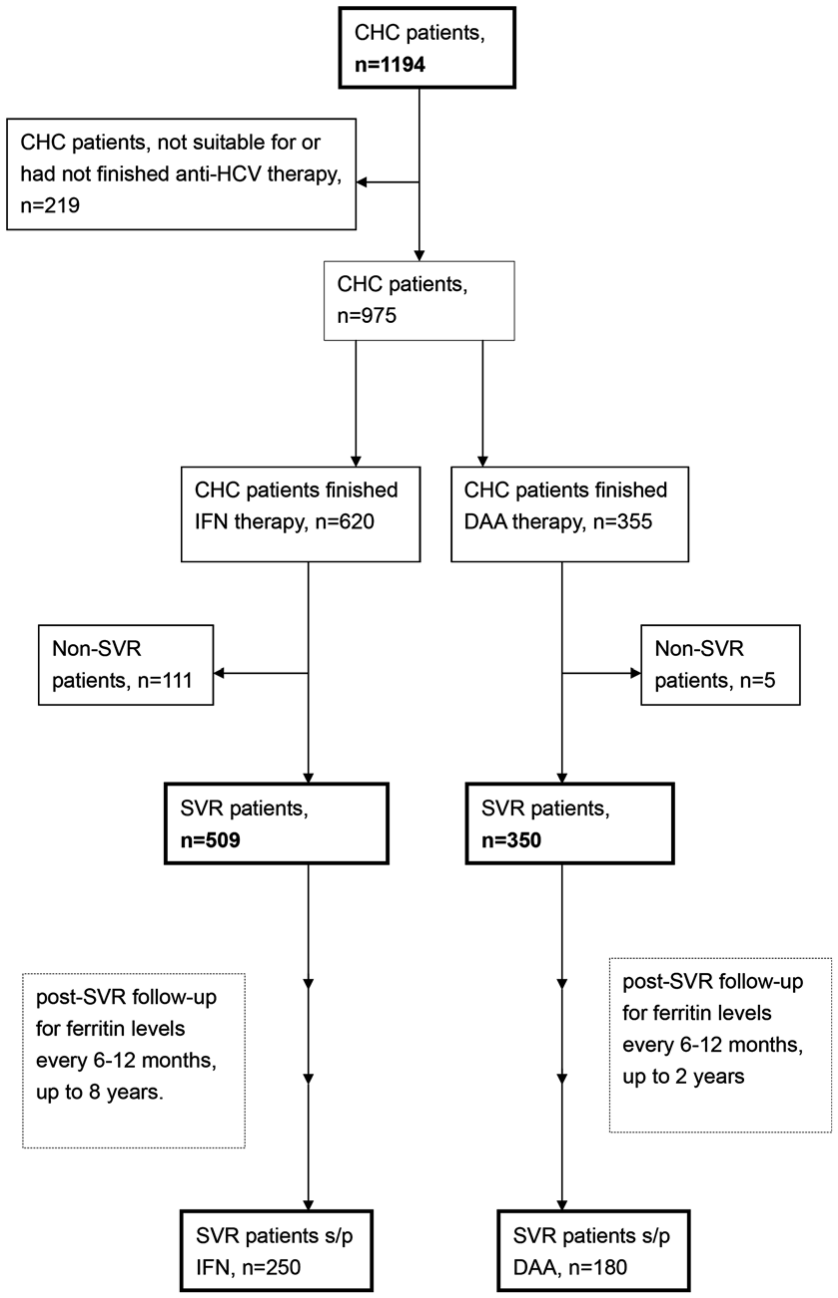
A schematic flow chart of the enrolled patients. *CHC* chronic hepatitis C virus infection, *IFN* interferon-based therapy, *DAA* direct-acting antiviral, *s/p* status post, *SVR* sustained virological response.

A schematic flow chart for all enrolled patients is shown in Fig. 1. In total, 1194 CHC patients were consecutively recruited at a Taiwan tertiary referral center between January 2010 and December 2019. Of the 1194 patients, 620 had completed a course of anti-HCV therapy with weight-based pegylated interferon-α-2b and ribavirin for either 24 or 48 weeks^22^, and 355 patients had completed a course of various combinations of DAAs (Supplementary Table 1). HCV-RNA levels, genotypes, and single-nucleotide polymorphisms of interferon-λ3 (IFNL3)-rs12979860 were assessed as previously described^22^. Several baseline factors, including sex, age, body mass index (BMI), HCV genotype, the presence of hepatic cirrhosis, levels of HCV-RNA, serum ferritin (normal range: male: 30–400 ng/mL; female: 13–150 ng/mL), iron, iron/total iron-binding capacity (Fe/TIBC) (i.e., transferrin saturation percentage), neutrophil–lymphocyte ratio (NLR), estimated glomerular filtration rate (eGFR), homeostatic model assessment of insulin resistance (HOMA-IR) index (fasting insulin [μU/mL] × fasting glucose [mmol/L]/22.5), total cholesterol (TC), triglycerides (TG), alanine aminotransferase (ALT), aspartate aminotransferase (AST), platelets and fibrosis-4 (FIB-4) index [age (years) × AST (U/L)/[PLT(10^9^/L) × ALT(U/L)^1/2^] were recorded in all patients. Biochemical tests were performed at the clinical pathology laboratories of the hospital using routine automated techniques in an automated clinical chemistry analyzer (Hitachi LST008). The diagnosis of hepatic steatosis was based on sonographic findings. The diagnosis of liver cirrhosis was made by earlier histologic findings or ultrasonographic findings compatible with cirrhosis and supplemented with esophageal or gastric varices, splenomegaly and/or thrombocytopenia, as described elsewhere^24^. For the CHC patients who completed the anti-HCV therapy, the aforementioned variable levels were evaluated 2 weeks before therapy and 24 weeks posttherapy. SVR was defined as undetectable levels of HCV-RNA 24 weeks after the completion of therapy. Ferritin levels were surveyed every 24–48 weeks posttherapy in SVR patients.

### Statistics

All statistical analyses were performed using the Statistical Package for Social Science (SPSS package version 21, SPSS Inc., Chicago, IL, USA) software. Continuous variables were analyzed using Student’s t-test, and categorical variables were analyzed using a chi-square test or Fisher’s exact test as appropriate. Nonparametric tests were performed where indicated. Multivariate linear or logistic regression models were used to assess relationships between various dependent and independent variables by adjusting for all the independent variables with a *p* value < 0.1 in univariate analyses. The collinearity of variables was determined by linear regression tests. A paired t-test was used to analyze the same variables before and after therapy within the same individuals. Statistical significance was assessed at the 5% level based on two-tailed tests of the null hypothesis^22,23^.

### Ethics approval

The study protocol conformed to the ethical guidelines of the 1975 Declaration of Helsinki and was approved by the local institutional review board (Chang Gung Memorial Hospital).

### Consent to participate

Written informed consent was obtained from each patient.

### Consent for publication

Not applicable.

## Results

### Baseline characteristics of the enrolled CHC patients

Of 1194 enrolled CHC patients with a mean age of 56.93 years, 627 (52.5%) were males, 679 (56.9%) were infected with genotype 1 HCV, and 240 (20.4%) had liver cirrhosis. The mean ferritin level was 386.0 ng/mL. Of the 1194 patients, 553 (46.3%) had high baseline ferritin levels. The comparisons between patients who underwent interferon-based therapy (n = 620) and those who underwent DAA therapy (n = 355) showed that the former were more frequently male, younger, infected with genotypes 1 and 2 HCV and had higher levels of ALT, eGFR, uric acid and hemoglobin; lower levels of NLR and FIB-4 index; and lower rates of hepatic steatosis (Table 1).

**Table 1:** Baseline characteristics of the 1194 CHC patients.

	All (n = 1194)	Interferon-based therapy (n = 620)	DAA therapy (n = 355)	*p* values*
Male, n (%)	627 (52.5)	353 (56.9)	166 (46.8)	0.001
Age (years)	56.93 ± 12.75	53.96 ± 11.72	60.33 ± 12.99	< 0.001
BMI (kg/m^2^)	24.80 ± 3.85	24.96 ± 3.76	24.80 ± 4.01	0.523
**HCV genotype**				
Genotype 1, n (%)	679 (56.9)	327 (52.7)	244 (63.1)	0.001
Genotype 2, n (%)	412 (34.5)	247 (39.8)	106 (29.9)	0.001
Genotype 3, n (%)	25 (2.1)	16 (2.6)	5 (1.4)	0.16
Log HCV RNA (logIU/mL)	6.00 ± 0.991	6.02 ± 1.05	6.03 ± 0.83	0.809
ALT (U/L)	91.89 ± 96.59	96.92 ± 98.24	81.69 ± 89.55	0.016
eGFR (mL/min/1.73 m^2^)	95.97 ± 38.40	102.73 ± 36.06	89.08 ± 39.86	< 0.001
TG (mg/dL)	103.54 ± 53.45	103.70 ± 52.77	103.97 ± 53.70	0.939
TC (mg/dL)	170.30 ± 33.49	171.52 ± 31.60	170.58 ± 33.78	0.666
HOMA-IR	3.23 ± 5.26	3.14 ± 4.45	3.24 ± 5.12	0.77
Uric acid (mg/dL)	5.86 ± 1.59	5.94 ± 1.55	5.71 ± 1.62	0.041
Ferritin (ng/mL)	386.0 ± 492.3	375.3 ± 434.1	381.7 ± 532.1	0.858
High ferritin, n (%)	553 (46.3)	296 (47.7)	164 (46.2)	0.853
Iron (μg/dL)	132.5 ± 56.5	138.9 ± 55.3	136.3 ± 58.7	2.678
Fe/TIBC (%)	0.404 ± 0.185	0.410 ± 0.181	0.40 ± 0.187	0.736
Hb (g/dL)	13.92 ± 1.85	14.30 ± 1.65	13.69 ± 1.92	< 0.001
NLR	1.75 ± 0.96	1.63 ± 0.84	1.88 ± 1.03	< 0.001
Platelet (10^3^/μL)	175.0 ± 66.3	176.9 ± 56.56	176.2 ± 72.2	0.884
Steatosis, n (%)	630 (52.8)	297 (47.9)	204 (57.5)	0.06
Liver cirrhosis, n (%)	243 (20.4)	136 (21.9)	84 (23)	0.38
FIB-4 index	3.39 ± 3.39	3.09 ± 3.23	3.86 ± 3.64	0.003
IFNL3-rs12979860 CC genotype, n (%)	1018 (85.3)	533 (85.9)	300 (84.5)	0.371

*CHC* chronic hepatitis C virus infection, *DAA* direct-acting antivirals, *BMI* body mass index, *HCV* hepatitis C virus, *RNA* ribonucleic acid, *ALT* alanine transaminase, *eGFR* estimated glomerular filtration rate, *TG* triglycerides, *TC* total cholesterol, *HOMA-IR* homeostatic model assessment for insulin resistance, *Fe/TIBC* serum Iron/total iron binding capacity, *Hb* hemoglobin, *NLR* neutrophil lymphocyte ratio, *FIB-4* fibrosis-4, *IFNL3* interferon-λ3.

*p values between CHC patients underwent interferon-based or DAA therapy.

### Factors associated with ferritin levels of CHC patients at baseline

At baseline, the variables including male sex, levels of ALT and TG, HOMA-IR, Fe/TIBC and CC genotype of IFNL3-rs12979860 were positively associated, while levels of eGFR and hemoglobin were negatively associated with ferritin levels among the 1194 CHC patients (Table 2). While we regarded high baseline ferritin levels as the dependent factor, the independent factors were age, ALT, eGFR, TGs, Fe/TIBC and fatty liver status (Supplementary Table 2). Of 620 patients who underwent interferon-based therapy, 509 achieved an SVR; of 355 patients who underwent DAA therapy, 350 achieved an SVR (Fig. 1). No differences in pretherapy ferritin levels were noted between SVR and non-SVR patients, regardless of therapy regimen (*p* = 0.912 for all patients, *p* = 0.417 for patients who underwent interferon-based therapy; *p* = 0.409 for patients who underwent DAA therapy). Furthermore, pretherapy ferritin levels were not associated with SVR, regardless of therapy regimens (*p* = 0.911 for all patients, *p* = 0.418 for patients who underwent interferon-based therapy; *p* = 0.353 for patients who underwent DAA therapy).

**Table 2:** Associations of ferritin levels in CHC patients at baseline.

Baseline factors	Univariate analyses		Multivariate analyses	
	95% CI of OR (OR)	*p* values	95% CI of OR (OR)	*p* values
Male, yes	98.3 ~ 225.5 (161.9)	< 0.001	2.935 ~ 130.3 (66.6)	0.04
Age (years)	0.667 ~ 5.741 (3.20)	0.013	− 4.366 ~ 1.196 (− 1.58)	0.264
BMI (kg/m^2^)	− 0.348 ~ 12.89 (4.71)	0.26		
HCV genotype	− 27.77 ~ 18.99 (− 4.39)	0.712		
Log HCV RNA (logIU/mL)	− 60.1 ~ 7.4 (− 26.3)	0.126		
ALT (U/L)	1.457 ~ 2.055 (1.756)	< 0.001	0.841 ~ 1.381 (1.111)	< 0.001
eGFR (mL/min/1.73 m^2^)	− 1.69 ~ − 1.007 (− 0.849)	0.048	− 1.97 ~ − 0.162 (− 1.066)	0.021
TG (mg/dL)	1.452 ~ 2.657 (2.055)	< 0.001	0.793 ~ 1.904 (1.349)	< 0.001
TC (mg/dL)	− 0.9 ~ 1.032 (0.066)	0.893		
HOMA-IR	2.054 ~ 15.057 (8.655)	0.01	0.806 ~ 12.073 (6.439)	0.025
Uric acid (mg/dL)	33.87 ~ 76.18 (55.0)	< 0.001	− 14.864 ~ 23.74 (4.438)	0.652
Fe/TIBC (%)	1428 ~ 1722 (1575)	< 0.001	1252 ~ 1565 (1409)	< 0.001
Hb (g/dL)	6.91 ~ 41.69 (24.3)	0.006	− 50.21 ~ − 13.5 (− 31.86)	0.001
NLR	− 23.8 ~ 43.4 (9.8)	0.567		
Platelet (10^3^/μL)	− 1.557 ~ − 0.588 (− 1.07)	< 0.001	− 3.46 ~ − 0.875 (0.265)	0.395
Steatosis, yes	− 8.2 ~ 118.5 (55.1)	0.188		
Liver cirrhosis, yes	− 107.7 ~ 52.4 (− 26.6)	0.519		
Fibrosis-4 index	16.4 ~ 34.8 (25.4)	< 0.001	− 3.20 ~ 20.42 (8.606)	0.153
IFNL3-rs12979860 CC genotype, yes	− 227.7 ~ − 34.7 (− 131.2)	0.008	− 181.9 ~ − 31.46 (− 106.2)	0.005

*CHC* chronic hepatitis C virus infection, *OR* odds ratio, *CI* confidence interval, *BMI* body mass index, *HCV* hepatitis C virus, *RNA* ribonucleic acid, *ALT* alanine transaminase, *eGFR* estimated glomerular filtration rate, *TG* triglycerides, *TC* total cholesterol, *HOMA-IR* homeostatic model assessment for insulin resistance, *Fe/TIBC* serum Iron/total iron binding capacity, *Hb* hemoglobin, *NLR* neutrophil lymphocyte ratio, *FIB-4* fibrosis-4, *IFNL3* interferon-λ3.

### Factors associated with ferritin levels of SVR patients at 24 weeks posttherapy

At 24 weeks posttherapy, the levels of ALT, TG, and TC and the ratio of Fe/TIBC were positively associated, while the levels of eGFR and DAA therapy were negatively associated, with the ferritin levels of SVR patients (Table 3).

**Table 3:** Associations of ferritin levels in SVR patients at 24 weeks posttherapy.

24-week post-therapy factors	Univariate analyses		Multivariate analyses	
	95% CI of OR (OR)	*p* values	95% CI of OR (OR)	*p* values
Male, yes	− 47.83 ~ 132.4 (42.29)	0.357		
Age, (years)	− 1.49 ~ 5.71 (2.1)	0.251		
BMI (kg/m^2^)	− 16.7 ~ 7.286 (− 4.7)	0.44		
ALT (U/L)	6.19 ~ 11.91 (9.052)	< 0.001	3.007 ~ 8.107 (5.557)	< 0.001
eGFR (mL/min/1.73 m^2^)	− 4.526 ~ − 1.821 (− 3.17)	< 0.001	− 3.9 ~ − 1.61 (− 2.76)	< 0.001
TG (mg/dL)	0.331 ~ 1.228 (0.779)	0.001	0.146 ~ 0.909 (0.527)	0.017
TC (mg/dL)	0.956 ~ 3.423 (2.189)	0.001	0.211 ~ 2.346 (1.278)	0.019
HOMA-IR	− 0.689 ~ 29.63 (14.47)	0.061	− 5.03 ~ 20.09 (7.53)	0.239
Uric acid (mg/dL)	33.59 ~ 91.36 (62.474)	< 0.001	− 7.11 ~ 40.52 (16.70)	0.169
Fe/TIBC (%)	1811 ~ 2338 (2075)	< 0.001	1521 ~ 2030 (1776)	< 0.001
Hb (g/dL)	− 34.76 ~ 18.93 (− 7.91)	0.563		
NLR	− 34.1 ~ 66.98 (16.45)	0.523		
Platelet (10^3^/μL)	− 2.06 ~ − 0.591 (− 1.29)	0.001	− 1.12 ~ 0.661 (− 0.234)	0.608
Steatosis, yes	− 177.8 ~ 86.3 (− 45)	0.495		
Liver cirrhosis, yes	− 77.0 ~ 256.8 (89.92)	0.29		
Fibrosis-4 index	− 2.56 ~ 42.1 (19.755)	0.083	− 4.36 ~ 47.8 (21.72)	0.102
IFNL3-rs12979860 CC genotype, yes	− 189.3 ~ 80.9 (− 54.2)	0.431		
Therapy (interferon = 1, DAA = 2)	− 352 ~ − 170 (− 261)	< 0.001	− 331 ~ − 161 (− 246)	< 0.001

*OR* odds ratio, *BMI* body mass index, *ALT* alanine transaminase, *eGFR* estimated glomerular filtration rate, *TG* triglycerides, *TC* total cholesterol, *HOMA-IR* homeostatic model assessment for insulin resistance, *Fe/TIBC* serum Iron/total iron binding capacity, *Hb* hemoglobin, *NLR* neutrophil lymphocyte ratio, *FIB-4* fibrosis-4, *IFNL3* interferon-λ3, *DAA* direct-acting antivirals.

**Figure 2:**
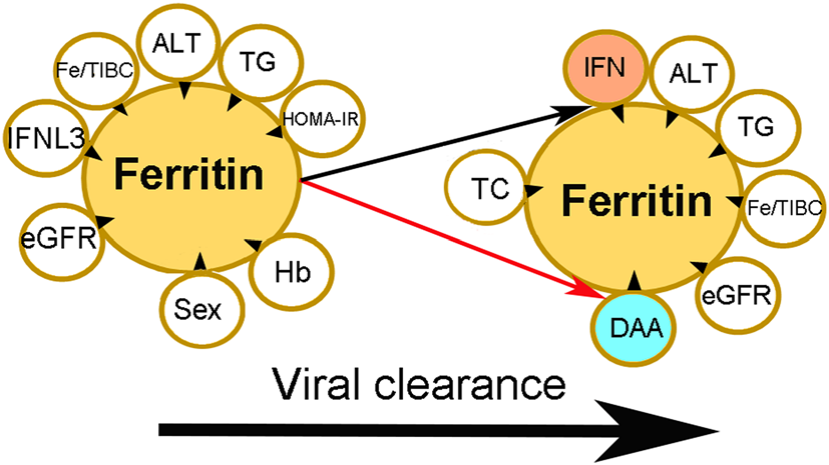
Associations between independent factors and ferritin levels at baseline and at 24 weeks post-anti-HCV therapy. The tips of the black arrowheads indicate dependent factors, and the bases of the black arrowheads indicate independent factors. *IFNL3* interferon λ3, *Fe/TIBC* iron/total iron-binding capacity, *ALT* alanine aminotransferase, *TG* triglycerides, *HOMA-IR* homeostasis model assessment-estimated insulin resistance, *eGFR* estimated glomerular filtration rate, *Hb* hemoglobin, *IFN* interferon-based therapy, *DAA* direct-acting antiviral, *TC* total cholesterol. Upward-facing black arrow: increased ferritin levels in patients who underwent interferon-based therapy; downward-facing red arrow: decreased ferritin levels in patients who underwent DAA therapy.

A summary of the associations (baseline and 24 weeks posttherapy) identified between independent factors and ferritin levels is shown in Fig. 2.

### Longitudinal alteration of ferritin levels in SVR patients

Among patients who received interferon-based therapy, compared with baseline, levels of ferritin increased at 24 weeks posttherapy, regardless of SVR [SVR (+) patients: 515 ± 672 vs. 365 ± 447 ng/mL, *p* < 0.001; SVR(−) patients: 896 ± 1281 vs. 418 ± 430 ng/mL, *p* = 0.001]. However, 24-week posttherapy levels of ferritin were higher in non-SVR patients than in SVR patients (896 ± 1281 vs. 515 ± 672 ng/mL, *p* = 0.001). Among patients who received DAA therapy and achieved an SVR (n = 350), the levels of ferritin decreased at 24 weeks posttherapy (227 ± 326 vs. 468 ± 632 ng/mL, *p* < 0.001), while among patients who received DAA therapy without an SVR (n = 5), the comparison between pretherapy and 24-week posttherapy levels of ferritin did not indicate a significant difference (180 ± 28.4 vs. 194 ± 129 ng/mL, *p* = 0.798). Among all SVR patients, lower levels of 24-week posttherapy ferritin were noted in patients who underwent DAA therapy than in those who underwent interferon-based therapy (227 ± 326 vs. 515 ± 672 ng/mL, *p* < 0.001) (Fig. 2). Furthermore, the percentage of patients with high ferritin levels was lower among SVR patients who underwent DAA therapy than among those who underwent interferon-based therapy (25.7% vs. 48.4%, *p* < 0.001).

**Figure 3:**
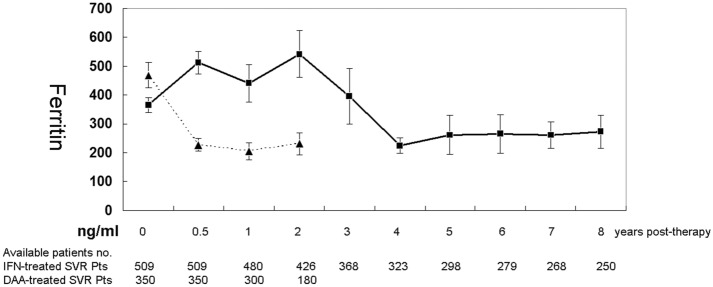
Longitudinal alteration in ferritin levels (mean ± standard error) in SVR patients following interferon (IFN)-based (solid line) and DAA (dashed line) therapies.

Longitudinally, as shown in Fig. 3, for SVR patients who underwent interferon-based therapy, compared with pretherapy levels, higher posttherapy ferritin levels were noted until 2 years posttherapy (*p* = 0.042), while the post- and pretherapy differences disappeared at 3 years posttherapy (*p* = 0.585), and the posttherapy levels were lower than pretherapy levels from 4 years (*p* = 0.004) to 8 years posttherapy (*p* = 0.012) (follow-up duration: mean ± standard deviation: 1736 ± 753 days, median: 1712 days, range 700–2920 days). For the SVR patients who underwent DAA therapy, posttherapy ferritin levels were lower than pretherapy ferritin levels from 24 weeks to 2 years posttherapy (*p* < 0.001) (final follow-up duration: mean ± standard deviation: 628 ± 196 days, median: 630 days, range 363–730 days). The ferritin levels in DAA-treated SVR patients remained steady beginning at 24 weeks posttherapy, as all the comparisons between posttherapy ferritin levels were nonsignificant (*p* values 0.471–0.646). Moreover, all the posttherapy ferritin levels of SVR patients who underwent DAA were lower than those of SVR patients who underwent interferon-based therapy with the same follow-up time (i.e., at 24 weeks and 1 and 2 years posttherapy) (Fig. 3, Supplementary Table 3).

## Discussion

The most compelling results of the current study are as follows:

(1) At baseline, sex, ALT, TG, HOMA-IR, eGFR, hemoglobin, Fe/TIBC and IFNL3-rs12979860 genotype were associated with ferritin levels of CHC patients. (2) At 24 weeks posttherapy, ALT, TG, TC eGFR, Fe/TIBC, and therapy regimen were associated with ferritin levels of SVR patients. (3) Among patients who received interferon-based therapy, the levels of ferritin increased at 24 weeks posttherapy, regardless of SVR. However, 24-week post-therapy ferritin levels were higher in non-SVR patients than in SVR patients. Among SVR patients who received DAA therapy, the levels of ferritin decreased at 24 weeks posttherapy. (4) During the 8-year follow-up, the trend of increased ferritin levels was reversed beginning at 4 years posttherapy in interferon-treated SVR patients; however, a stable trend of decreased ferritin levels remained in DAA-treated SVR patients starting at 24 weeks posttherapy.

It has been proposed that HCV controls iron both by intracellular iron sequestration and intercellular iron mobilization via ferritin, as a means toward enhanced replication^3^. However, consistent with a previous study^13^, no significant correlations were identified between HCV viral load and any iron markers. The factors consistently associated with ferritin levels both pretherapy in CHC patients and at 24 weeks posttherapy in SVR patients, such as ALT, TG, eGFR, and Fe/TIBC, exhibited fundamental links with ferritin, regardless of HCV infection. For example, ferritin levels > 1.5 times higher than normal are usually seen in patients with a > 6 month history of elevated ALT^25^, high TG levels are strongly associated with ferritin levels^26^, every 100 μg/L increase in ferritin levels was correlated with 0.26 mL/min per 1.73 m^2^ decrease in eGFR^27^, and the positive association between ferritin levels and transferrin saturation (Fe/TIBC) reflects the correlation between iron store and iron availability^28^. In contrast, the pretherapy-only factors (sex, HOMA-IR, hemoglobulin and IFNL3-rs12979860 genotype) and posttherapy-only factors (TC and anti-HCV therapy) suggested potential links, direct or indirect, between HCV infection and ferritin levels. Consistently, higher hepatic iron concentrations were observed in male CHC patients^29^; the connection between HOMA-IR and ferritin levels in CHC patients has been reported^30^ and may correlate with the grade of hepatic iron deposition^31,32^; the negative association between hemoglobin and ferritin levels among CHC patients seemed to reflect the links between fibrosis (portal hypertension subsequent to hepatic fibrosis might lead to variceal bleeding with anemia and then low hemoglobin levels) and ferritin levels, as elevated serum ferritin levels are independently associated with advanced liver fibrosis in CHC patients^12^. Of note, it is a novel finding that the IFNL3-rs12979860 CC genotype was negatively associated with baseline ferritin levels, which echoes previous studies revealing that baseline ferritin levels were independently associated with poor response to interferon-based therapy^12,33,34^; the association might occur through advanced hepatic fibrosis^12,35^, hepatic steatosis^12,36,37^ or high necroinflammatory activity^35^. However, the baseline ferritin levels were not associated with SVR among interferon-treated patients in the current study, and a prevalent IFNL3-rs12979860 CC genotype in Taiwan^22,23^ might blunt the impact of baseline ferritin levels on SVR. Interestingly, HCV nonstructural proteins upregulate the ferritin heavy chain, which in turn inhibits apoB-100 secretion, and serum ferritin and apoB-100 concentrations are inversely correlated in HCV-infected patients^38^. The positive association between ferritin and TC among SVR patients might reflect the reversal of HCV-associated inhibition of the secretion of apoB-100, the main apolipoprotein of TC, after HCV clearance. Given that sex, HOMA-IR, TC, hemoglobin and IFNL3-rs12979860 genotype were HCV-related factors associated with serum ferritin levels, special caution is demanded in male CHC patients with high HOMA-IR and TC but low Hb levels and an IFNL3-rs12979860 non-CC genotype due to the potential poor prognosis linked with high ferritin levels. Namely, the demographic, genetic and metabolic profiles, in particular lipid and glucose parameters, might affect the outcomes of CHC patients by interfering with ferritin levels.

Therapy regimens also affect ferritin levels in CHC patients. Serum ferritin levels were significantly decreased at the end of therapy^39^, at 12 weeks^40^, 24 weeks^41^, or up to 1 year^42^ from baseline in SVR patients following DAA therapy. Since ferritin might work as an acute phase protein and reflect hepatic injury, it is conceivable that ferritin levels decreased after SVR in CHC patients following DAA therapy. Compared with baseline levels, the lower ferritin levels persisted up to 2 years posttherapy among DAA-treated SVR patients in the current study, suggesting a long-term improvement in iron homeostasis^20^. On the other hand, although iron overload improved after SVR in CHC patients who underwent interferon-based therapy^43^, how ferritin levels evolved in these patients remains inconclusive, as ferritin levels had been reported to increase^44^, decrease^45,46^ or remain steady^42^ in studies with case numbers ranging from 73 to 191. Based on a cohort of 620 CHC patients who underwent interferon-based therapy, our study demonstrated that the levels of ferritin increased at 24 weeks posttherapy, rather than decreased, regardless of SVR, although the 24-week posttherapy ferritin levels were lower in the SVR patients than in the non-SVR patients. The fact that ferritin levels increased at 24 weeks posttherapy in CHC patients who underwent interferon monotherapy regardless of viral clearance^47^ suggests that interferon per se increases ferritin levels. On the other hand, ribavirin-induced hemolysis was shown to significantly increase serum ferritin levels, intrahepatic iron deposition and liver fibrosis in renal transplant patients receiving ribavirin monotherapy^48^. Additionally, ribavirin-induced hemolysis floods hepatocytes and Kupffer cells with heme, which is metabolized and detoxified by heme oxygenase-1 to carbon monoxide, biliverdin and free iron, which induces ferritin^49^. Together, pegylated interferon and ribavirin of interferon-based therapy might synergistically increase serum ferritin levels in CHC patients. This idea explained why both SVR and non-SVR patients treated with interferon-based therapy had increased 24-week posttherapy ferritin levels. Surprisingly, it took at least 4 years to eliminate interferon- and ribavirin-related biases, as the trend of increased ferritin levels was not reversed until 4 years posttherapy. Thus, compared with interferon-based therapy, DAA therapy is not only more effective, safer and more tolerable^50^ but also leads to a less vulnerable iron homeostasis status, evidenced by earlier reversal of high ferritin levels after SVR.

Given that most non-SVR patients following interferon-based therapy had received further therapeutic courses of DAA, their long-term post-interferon-based-therapy ferritin levels cannot be acquired. Thus, the major limitation of the current study is that the long-term posttherapy ferritin levels between the SVR and non-SVR patients following interferon-based therapy cannot be compared. However, as mentioned^21^, the comparisons between the pre- and posttherapy ferritin levels in the same individuals might be a better alternative to compare the levels between SVR and non-SVR patients in terms of eliminating the individual biases.

In summary, ALT, TG, eGFR, and Fe/TIBC were HCV-unrelated factors associated with ferritin levels, while sex, HOMA-IR, TC, hemoglobin and IFNL3-rs12979860 genotype were HCV-related factors associated with serum ferritin levels. During a follow-up of 8 years, in interferon-treated SVR patients, the trend of increased posttherapy ferritin levels was not reversed until 4 years posttherapy. In SVR patients treated with DAA therapy, ferritin levels decreased at 24 weeks posttherapy and remained stable afterward. These specific evolutions and associations of ferritin levels indicated that a tailored follow-up protocol for hyperferritinaemia and associated complications in CHC patients with SVR needs to be conducted according to antiviral regimens.

## Supplementary information

Supplementary Information.

## Data Availability

The data that support the findings of this study are available on request from the corresponding author (MLC).
