# Supplementary material for: Evolution of ferritin levels in hepatitis C patients treated with antivirals
Source: Sci Rep. 2020 Nov 12;10:19744. doi: 10.1038/s41598-020-76871-z (PMC7661708; doi:10.1038/s41598-020-76871-z)
Supplement: Supplementary file 1 — Supplementary Information. [file 41598_2020_76871_MOESM1_ESM.doc]

**Evolution of ferritin levels in hepatitis C patients treated with antivirals**

**Ming-Ling Chang, Jing-Hong Hu, Ching-Hao Yen, Kuan-Hsing Chen, Chia-Jung Kuo, Ming-Shyan Lin, Cheng-Han Lee, Shiang-Chi Chen, Rong-Nan Chien**

**Supplementary Table 1. Various DAA combinations used** in the study.

| Genotype 1 |  | Genotype 2 |  | Genotype 3 |  | Genotype 4 |  | Genotype 5 |  | Genotype 6 |  |
| --- | --- | --- | --- | --- | --- | --- | --- | --- | --- | --- | --- |
| DAA | D(W) | DAA | D(W) | DAA | D(W) | DAA | D(W) | DAA | D(W) | DAA | D(W) |
| Asunaprevir (100 mg, bid) with Daclatasvir (60 mg, qd) | 24 | Sovaldi (Sofosbuvir 400 mg qd) with Ribavirin | 12 |  |  |  |  |  |  |  |  |
| Viekirax (Ombitasvir 12.5mg/Paritaprevir 75mg/Ritonavir 50 mg qd) with Exviera (250 mg bid) | 12-24 |  |  |  |  |  |  |  |  |  |  |
| Zepatier (Elbasvir 50 mg/Grazoprevir 100 mg, qd) | 12-16 |  |  |  |  |  |  |  |  |  |  |
| Harvoni (Ledipasvir 90 mg /Sofosbuvir 400 mg qd) with/without Ribavinin (800-1400 mg/day, in two divided doses | 12 | Harvoni (Ledipasvir 90 mg /Sofosbuvir 400 mg qd) with/without Ribavinin (800-1400 mg/day, in two divided doses | 12 |  |  | Harvoni (Ledipasvir 90 mg /Sofosbuvir 400 mg qd) with/without Ribavinin (800-1400 mg/day, in two divided | 12 | Harvoni (Ledipasvir 90 mg /Sofosbuvir 400 mg qd) with/without Ribavinin (800-1400 mg/day, in two divided | 12 | Harvoni (Ledipasvir 90 mg /Sofosbuvir 400 mg qd) with/without Ribavinin (800-1400 mg/day, in two divided | 12 |
| Mavyret (glecaprevir 100mg/pibrentasvir 40mg, 3# qd) | 8-16 | Mavyret (glecaprevir 100mg/pibrentasvir 40mg, 3# qd) | 8-12 | Mavyret (glecaprevir 100mg/pibrentasvir 40mg, 3# qd) | 8-16 | Mavyret (glecaprevir 100mg/pibrentasvir 40mg, 3# qd) | 8-12 | Mavyret (glecaprevir 100mg/pibrentasvir 40mg, 3# qd) | 8-12 | Mavyret (glecaprevir 100mg/pibrentasvir 40mg, 3# qd) | 8-12 |
| Epclusa (sofosbuvir 400 mg/velpatasvir 100 mg qd) with or without Ribavirin | 12 | Epclusa (sofosbuvir 400 mg/velpatasvir 100 mg qd) with or without Ribavirin | 12 | Epclusa (sofosbuvir 400 mg/velpatasvir 100 mg qd) with or without Ribavirin | 12 | Epclusa (sofosbuvir 400 mg/velpatasvir 100 mg qd) with or without Ribavirin | 12 | Epclusa (sofosbuvir 400 mg/velpatasvir 100 mg qd) with or without Ribavirin | 12 | Epclusa (sofosbuvir 400 mg/velpatasvir 100 mg qd) with or without Ribavirin | 12 |

DAA: direct-acting antiviral agent; D: duration: W: weeks.

**Supplementary Table 2. Associations of high** ferritin levels in CHC patients at baseline

| Baseline factors | Univariate analyses | | Multivariate analyses | |
| --- | --- | --- | --- | --- |
|  | 95% CI of OR (OR) | *p* values | 95% CI of OR (OR) | *p* values |
| Male, yes | 0.491~0.806 (0.629) | 0.379 |  |  |
| Age (years) | 1.016~1.037 (1.026) | <0.001 | 1.002~1.037 (1.019) | 0.027 |
| BMI (kg/m^2^) | 0.986~1.047 (1.016) | 0.304 |  |  |
| HCV genotype | 0.931~1.11 (1.016) | 0.719 |  |  |
| Log HCV RNA (logIU/mL) | 0.81~1.065 (0.929) | 0.29 |  |  |
| ALT (U/L) | 1.007~1.012 (1.010) | <0.001 | 1.006~1.012 (1.009) | <0.001 |
| eGFR (mL/min/1.73 m^2^) | 0.992~0.999 (0.995) | 0.005 | 0.988~0.999 (0.993) | 0.014 |
| TG (mg/dL) | 1.001~1.006 (1.004) | 0.002 | 1.001~1.008 (1.004) | 0.017 |
| TC (mg/dL) | 0.997~1.004 (1.000) | 0.826 |  |  |
| HOMA-IR | 0.993~1.053 (1.022) | 0.137 |  |  |
| Uric acid (mg/dL) | 1.008~1.186 (1.093) | 0.032 | 0.811~1.014 (0.907) | 0.087 |
| Fe/TIBC (%) | 94.8~636.0 (245.6) | <0.001 | 48.1~457.3 (148.4) | <0.001 |
| Hb (g/dL) | 0.962~1.10 (1.029) | 0.403 |  |  |
| NLR | 0.732~0.951 (0.834) | 0.007 | 0.798~1.136 (0.952) | 0.588 |
| Platelet (10^3^/uL) | 0.993~0.997 (0.995) | <0.001 | 0.993~1.000 (0.997) | 0.089 |
| Steatosis, yes | 1.069~1.785 (1.382) | 0.013 | 1.024~2.018 (1.438) | 0.036 |
| Liver cirrhosis, yes | 0.686~1.286 (0.939) | 0.694 |  |  |
| Fibrosis-4 index | 1.037~1.125 (1.081) | <0.001 | 0.868~1.017 (0.94) | 0.129 |
| IFNL3-rs12979860 CC genotype, yes | 0.413~0.883 (0.604) | 0.009 | 0.496~1.221 (0.778) | 0.276 |

CHC: chronic hepatitis C virus infection; CI: confidence interval; OR: odds ratio; BMI: body mass index; HCV: hepatitis C virus; RNA: ribonucleic acid; ALT: alanine transaminase; eGFR: estimated glomerular filtration rate; TG: triglycerides; TC: total cholesterol; HOMA-IR: homeostatic model assessment for insulin resistance; Fe/TIBC: serum Iron/total iron binding capacity; Hb: hemoglobin:; NLR: neutrophil lymphocyte ratio; FIB-4: fibrosis-4; IFNL3; interferon-λ3.

**Supplementary Table 3. Comparisons of posttherapy ferritin levels between SVR patients underwent interferon-based and DAA-based therapy**.

|  | Baseline | | 0.5 year post-therapy | | 1 years post-therapy | | 2 years post-therapy | |
| --- | --- | --- | --- | --- | --- | --- | --- | --- |
|  | Mean+/-SE | *p* values | Mean+/-SE | *p* values | Mean+/-SE | *p* values | Mean+/-SE | *p* values |
| IFN group | 365.4+/-25.6 | 0.09 | 488.1+/-32.5 | <0.001 | 450.8+/-26.3 | 0.001 | 520+/-38.8 | 0.002 |
| DAA group | 441+/-38.9 |  | 227.6+/-22.5 |  | 210+/-20.3 |  | 229+/-23.5 |  |

SD: standard error; IFN: interferon-based therapy; DAA: direct-acting antiviral agent
